# Evaluation of the Community Support Programme applied at the Intensive Psychiatric Rehabilitation Unit at the Parc Sanitari Sant Joan de Déu, Sant Boi

**DOI:** 10.1192/j.eurpsy.2022.1949

**Published:** 2022-09-01

**Authors:** M. Contel, M. Laszlo, L. Prats, E. Vadillo, J. Guillén

**Affiliations:** 1 Parc Sanitari San Joan de Déu, Intensive Psychiatric Rehabilitation Unit, Sant Boi de Llobregat, Spain; 2 Parc Sanitari Sant Joan de Déu, Intensive Psychiatric Rehabilitation Unit, Sant Boi de Llobregat, Spain

**Keywords:** community support programme, psychiatry rehabilitation, outcomes, severe mental illness

## Abstract

**Introduction:**

Our hospital has chosen a model that goes beyond long-term hospital inpatient care to a community support for people with severe and persisting mental illnesses. This programme is called Community Support Programme (CSP) and focuses mainly on connecting patients who are being discharged from long-term hospitalization to a community based rehabilitation service.

**Objectives:**

To evaluate the effectiveness of the CSP among people with severe and persisting mental illness, as a method to support the positive outcomes and reduce the use of hospital resources after a discharge.

**Methods:**

This is a retrospect, observational descriptive study. We reviewed 55 cases between 2017 and May of 2021. We analyzed demographic information, diagnosis, duration of stay in the CSP, number of hospitalizations before and after the program, number of emergency visits before and after this program and what kind of community rehabilitation services are connected after the discharge.

**Results:**

We found 58,2% male and 41,8% female. The main diagnoses were schizophrenia; schizoaffective and bipolar disorder. Before the CSP 85.4% of the patients had been hospitalized, and 76% had attended in a psychiatric emergency unit. After the discharge 36,36% required hospitalization, and 40% visited the psychiatric emergencies units. 54,54% of patients didn’t require hospital resources after their discharge from CSP.

**Conclusions:**

The results suggest that the CSP helps to avoid hospitalization, reduce the use of hospital resources and drop outs. It helps the transition from hospitalization to a community based rehabilitation service.

**Disclosure:**

No significant relationships.

